# Hot Electrons Induced by Localized Surface Plasmon Resonance in Ag/g-C_3_N_4_ Schottky Junction for Photothermal Catalytic CO_2_ Reduction

**DOI:** 10.3390/polym16162317

**Published:** 2024-08-16

**Authors:** Peng Jiang, Kun Wang, Wenrui Liu, Yuhang Song, Runtian Zheng, Lihua Chen, Baolian Su

**Affiliations:** 1 State Key Laboratory of Advanced Technology for Materials Synthesis and Processing, Wuhan University of Technology, Wuhan 430070, China; 2 Laboratory of Inorganic Materials Chemistry, University of Namur, B-5000 Namur, Belgium

**Keywords:** Ag/g-C_3_N_4_, Schottky junction, photothermal catalytic CO_2_ reduction, hot electrons, plasmonic metal/polymer semiconductor

## Abstract

Converting carbon dioxide (CO_2_) into high-value-added chemicals using solar energy is a promising approach to reducing carbon dioxide emissions; however, single photocatalysts suffer from quick the recombination of photogenerated electron–hole pairs and poor photoredox ability. Herein, silver (Ag) nanoparticles featuring with localized surface plasmon resonance (LSPR) are combined with g-C_3_N_4_ to form a Schottky junction for photothermal catalytic CO_2_ reduction. The Ag/g-C_3_N_4_ exhibits higher photocatalytic CO_2_ reduction activity under UV-vis light; the CH_4_ and CO evolution rates are 10.44 and 88.79 µmol·h^−1^·g^−1^, respectively. Enhanced photocatalytic CO_2_ reduction performances are attributed to efficient hot electron transfer in the Ag/g-C_3_N_4_ Schottky junction. LSPR-induced hot electrons from Ag nanoparticles improve the local reaction temperature and promote the separation and transfer of photogenerated electron–hole pairs. The charge carrier transfer route was investigated by in situ irradiated X-ray photoelectron spectroscopy (XPS). The three-dimensional finite-difference time-domain (3D-FDTD) method verified the strong electromagnetic field at the interface between Ag and g-C_3_N_4_. The photothermal catalytic CO_2_ reduction pathway of Ag/g-C_3_N_4_ was investigated using in situ diffuse reflectance infrared Fourier transform spectra (DRIFTS). This study examines hot electron transfer in the Ag/g-C_3_N_4_ Schottky junction and provides a feasible way to design a plasmonic metal/polymer semiconductor Schottky junction for photothermal catalytic CO_2_ reduction.

## 1. Introduction

Gradually increasing carbon dioxide (CO_2_) content in the ambient atmosphere has resulted in the greenhouse effect, which has caused serious ecological damage and climate change [1,2,3,4]. The conversion of carbon dioxide into high-value chemicals and energetic fuels to achieve carbon neutrality could contribute to a new balance of the carbon cycle. This could not only reduce carbon dioxide emissions, but also alleviate the energy crisis. It is a promising way to convert carbon dioxide into high-value-added chemicals and energy-containing fuels through solar energy [5,6,7,8]. Photocatalysis combined with the additional driving force provided by the thermal effect is beneficial to increasing CO_2_ reduction and value-added product generation. Photothermal catalysis integrating photochemical and thermochemical processes is considered a promising technology to convert solar energy into chemical energy [9,10,11,12].

Compared with the thermocatalytic process, which consumes considerable energy, photothermal catalysis induced by the photothermal effect is energy-saving and environmentally friendly [13,14,15,16]. Photothermal catalytic CO_2_ reduction could not only improve the utilization of solar energy but also promote the improvement of catalytic efficiency. Heat generated by the photothermal effect promotes the photocatalytic CO_2_ reaction process through thermochemical pathways, and photon energy also significantly contributes to reaction activity, resulting in a synergistic effect of thermochemical and photochemical pathways that is different to simply adding these two pathways together [17,18]. Photothermal catalysts dissipate absorbed photon energy into thermal energy under light irradiation, which is conducive to the transfer of charge carriers and improves catalytic CO_2_ reaction activity [19,20]. Hot electrons are generated at photothermal catalysts upon irradiation and then participate in the photocatalytic CO_2_ reaction. Photothermal catalysis enhances the reaction activity because of the synergy between the photochemical process and the thermochemical process, thereby exhibiting excellent photothermal catalytic CO_2_ reduction performance [21,22].

Single photocatalysts show unsatisfactory photocatalytic CO_2_ reaction performances due to the rapid recombination of photogenerated electrons and holes. It is necessary to address the issue of rapid recombination of photogenerated electron–hole pairs to improve the photocatalytic CO_2_ reaction performance. Designing plasmonic metal/semiconductor Schottky junction materials can improve the efficiency of spatial charge separation, thereby suppressing photogenerated electron and hole pair recombination and enhancing photocatalytic CO_2_ reaction performance [23,24,25,26]. Additionally, the plasmonic metal/semiconductor Schottky junction can be used for photothermal catalytic CO_2_ reduction due to the photothermal effect induced by the localized surface plasmon resonance (LSPR) of plasmonic metal [27,28]. Therefore, designing plasmonic metal/semiconductor Schottky junction materials has become a promising solution to realize photothermal catalytic CO_2_ reduction.

As a promising polymer semiconductor, graphitic carbon nitride (g-C_3_N_4_) has attracted increasing attention in photocatalytic CO_2_ reduction due to its low cost, high thermal and chemical stability, easy preparation process, semiconductivity, and appropriate band gap [29,30,31,32]. However, g-C_3_N_4_ exhibits low photocatalytic CO_2_ reduction activity owing to its rapid recombination of photogenerated electrons and holes and poor photoreduction ability. Noble metals can enhance the optical absorption of their adjacent semiconductors. Gold, silver, palladium, and other noble metals featuring LSPR can decay through the radiative pathway to create local thermal heating and electromagnetic field enhancements near the particle and generate hot electrons in a nonradiative way [33,34,35,36,37]. Hot electrons with high energy overstep the Schottky barrier at the interfaces of the plasmonic metal/polymer semiconductor Schottky junction. They can directly inject into the conduction band of polymer semiconductors to drive the surface photocatalytic CO_2_ reduction [38,39]. Thus, the coupling of g-C_3_N_4_ and noble metals is expected to form a plasmonic metal/polymer semiconductor Schottky junction which can promote the separation and transfer of photogenerated electrons and holes, thereby exhibiting excellent photothermal catalytic CO_2_ reduction performance.

Herein, the Ag/g-C_3_N_4_ Schottky junction featuring LSPR is designed for photothermal catalytic CO_2_ reduction. Compared with g-C_3_N_4_, the Ag/g-C_3_N_4_ Schottky junction photocatalyst exhibits higher CH_4_ (10.44 µmol·h^−1^·g^−1^) and CO (88.79 µmol·h^−1^·g^−1^) evolution rates with enhanced local reaction temperatures. The Ag/g-C_3_N_4_ Schottky junction is designed for photothermal catalytic CO_2_ reductions. Still, the underlying photothermal catalytic CO_2_ reduction mechanism, including hot electron transfer routes for photothermal catalytic CO_2_ reduction and the reduction of CO_2_ to CH_4_ and CO pathways, is unknown. Therefore, in situ irradiated X-ray photoelectron spectroscopy (XPS) was employed to investigate electron transfer in the Ag/g-C_3_N_4_ Schottky junction, and in situ diffuse reflectance infrared Fourier transform spectroscopy (DRIFTS) spectra were applied to detect the photothermal catalytic CO_2_ reduction pathway over the Ag/g-C_3_N_4_ photocatalyst. Three-dimensional finite-difference time-domain (3D-FDTD) verified the strong electromagnetic field at the interface between Ag and g-C_3_N_4_. Efficient hot electron transfer in the Ag/g-C_3_N_4_ Schottky junction improves the local reaction temperature and promotes the separation and transfer of photogenerated charge carriers, thereby enhancing photocatalytic CO_2_ reduction performance. This study reveals the efficient electron transfer and photothermal CO_2_ reduction mechanism of the Ag/g-C_3_N_4_ Schottky junction and provides a feasible way to design a plasmonic metal/polymer semiconductor Schottky junction for photothermal catalytic CO_2_ reduction.

## 2. Materials and Methods

### 2.1. Materials

Melamine (C_3_H_6_N_6_, 99%) was obtained from Shanghai Aladdin Biochemical Technology Co., Ltd., Shanghai, China. Silver nitrate (AgNO_3_, 99.99%) was bought from China National Pharmaceutical Group Chemical Reagent Co., Ltd., Beijing, China. Deionized water was purchased from Sinopharm Chemical Reagent Co., Ltd, Beijing, China. All chemicals were used without further purification.

### 2.2. Synthesis of Ag/g-C_3_N_4_

An amount of 5 g of melamine was put into the crucible, and then calcinated at 500 °C for 6 h with a ramp rate of 2 °C min^−1^ in a muffle furnace. After cooling to room temperature, bulk g-C_3_N_4_ was obtained. The bulk g-C_3_N_4_ was ground with a mortar to obtain the g-C_3_N_4_ (CN) powder. Next, 1 g of g-C_3_N_4_ powder and 0.05 g of AgNO_3_ were dispersed into 30 mL of deionized water under magnetic stirring for 5 h. Then, the above-obtained suspension was irradiated by a Xenon lamp under magnetic stirring for 6 h. Finally, the mixture was dried in a baking oven at 60 °C for 24 h to obtain Ag/CN-1. Meanwhile, 1 g of g-C_3_N_4_ powder and 0.1g of AgNO_3_ were dispersed into 30 mL of deionized water under magnetic stirring for 5 h. Then, the above-obtained suspension was irradiated by a Xenon lamp under magnetic stirring for 6 h. Finally, the mixture was dried in a baking oven at 60 °C for 24 h to obtain Ag/CN-2.

### 2.3. Photocatalytic CO_2_ Reduction

We added 50 mg of catalyst (CN, Ag/CN-1, and Ag/CN-2) into a 200 mL stainless steel reactor with an optical quartz window at the top. The reactor was first vacuumed, and then H_2_ and CO_2_ were introduced into the stainless steel reactor at a volume ratio of 4:1 for an hour to blow out the air in the reactor. A 300 W Xenon lamp (PLS-SXE300, Beijing PerfectLight, Beijing, China) with a filter (AM 1.5 G, Ceaulight Technology Co., Ltd., Beijing, China) was employed to simulate solar illumination for photocatalytic CO_2_ reduction with about 100 mW·cm^−2^. A Xenon lamp irradiated the reactor for 3 h. During the photocatalytic CO_2_ reduction process, the gaseous mixture was periodically sampled from the stainless steel reactor every 0.5 h and analyzed using gas chromatography (GC 9790 II, Fuli Instruments, Wenling, China).

## 3. Results and Discussion

### 3.1. Microstructure and Physical Properties Analysis

**Figure 1:**
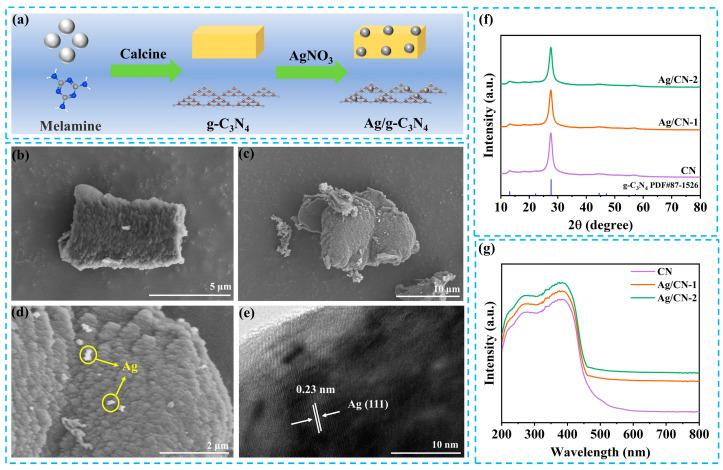
(**a**) Scheme of the preparation of Ag/g-C_3_N_4_; FSEM of (**b**) g-C_3_N_4_ and (**c**,**d**) Ag/g-C_3_N_4_; (**e**) high-resolution TEM (HRTEM) images of Ag/g-C_3_N_4_; (**f**) XRD patterns of g-C_3_N_4_, TiO_2_ and g-C_3_N_4_/TiO_2_; (**g**) UV-vis DRS of CN, Ag/CN-1 and Ag/CN-2.

The preparation process of Ag/g-C_3_N_4_ is illustrated in Figure 1a. Firstly, melamine is calcined to become g-C_3_N_4_ at high temperatures. Afterward, the Ag nanoparticles are coated on the surface of the g-C_3_N_4_ to form the Ag/g-C_3_N_4_ Schottky junction. The field-emission scanning electron microscopy (FSEM) was employed to study the microstructure of the photocatalysts. As shown in Figure 1b, the g-C_3_N_4_ shows a bulk structure with a size of 5 µm. As Figure 1c,d shows, the Ag nanoparticles are highly dispersed on the surface of the g-C_3_N_4_, indicating the loading of Ag nanoparticles on the g-C_3_N_4_. The high-resolution TEM (HRTEM) image shows that the lattice-fringe spacing of 0.23 nm is indexed into the (111) plane of Ag (Figure 1e), implying the existence of Ag nanoparticles in the Ag/g-C_3_N_4_ Schottky junction.

X-ray powder diffraction (XRD) patterns were applied to investigate the phase structures of the photocatalysts. As shown in Figure 1f, all the CN, Ag/CN-1, and Ag/CN-2 peaks correspond to the typical diffraction pattern of g-C_3_N_4_ (PDF#87-1526) [29,40,41]. The crystallinity percentages of different g-C_3_N_4_-based photocatalysts are the same [42]. The diffraction peaks of Ag are not observed in the CN, Ag/CN-1, and Ag/CN-2 samples because of the low content of Ag. The above results suggest that the Ag/g-C_3_N_4_ Schottky junction photocatalyst was successfully prepared. The nitrogen adsorption–desorption isotherms of CN, Ag/CN-1, and Ag/CN-2 display typical type IV isotherms with an H_3_ hysteresis loop (Figure S1). Brunauer–Emmett–Teller surface areas (S_BET_) of CN, Ag/CN-1, and Ag/CN-2 are 13.5, 12.2, and 11.4 m^2^·g^−1^, respectively (Table S1). The light absorption abilities of CN, Ag/CN-1, and Ag/CN-2 were measured using UV-vis diffuse reflectance spectra (UV-vis DRS spectra). In Figure 1g, the light adsorption intensities of Ag/CN-1 and Ag/CN-2 are higher than those of CN, especially in the visible light region. Notably, compared with the CN, the absorption edge of Ag/CN-1 and Ag/CN-2 shows no shift. The result of UV-vis DRS spectra suggests that loading Ag nanoparticles on the g-C_3_N_4_ improves visible light adsorption ability, which is advantageous for photocatalytic CO_2_ reduction.

### 3.2. Photothermal Catalytic CO_2_ Reduction Performance

**Figure 2:**
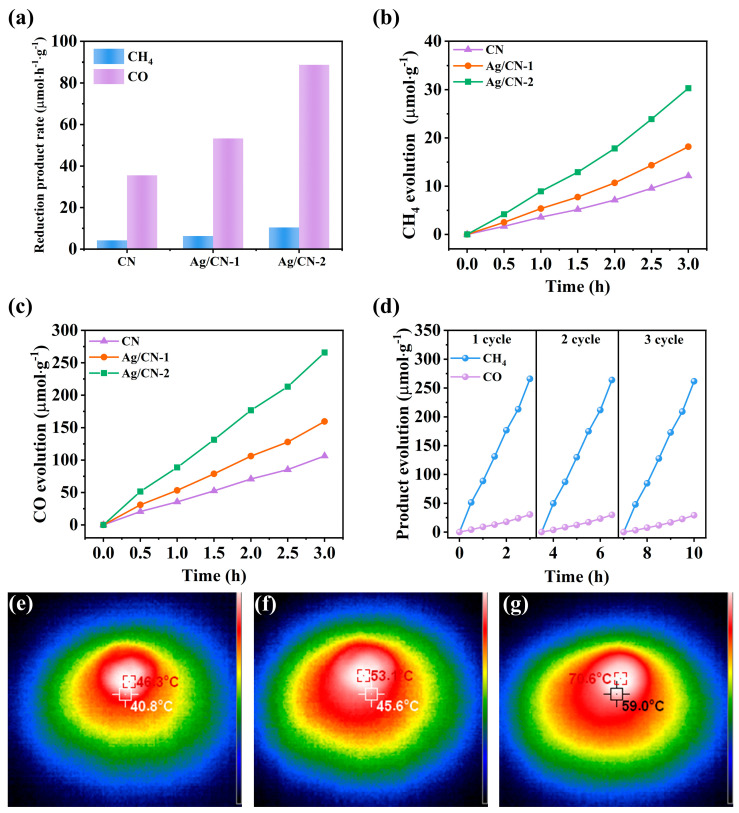
(**a**) Products evolution rate via photocatalytic CO_2_ reaction; (**b**) time-dependent CH_4_ evolution; (**c**) time-dependent CO evolution of CN, Ag/CN-1, and Ag/CN-2; (**d**) photocatalytic stability test of Ag/CN-2; infrared thermograms of (**e**) CN, (**f**) Ag/CN-1, and (**g**) Ag/CN-2 under the Xe lamp irradiation.

The photothermal catalytic CO_2_ reduction performances of CN, Ag/CN-1, and Ag/CN-2 photocatalysts were measured under UV-vis light. As shown in Figure 2a, CH_4_ and CO are the major photoreduction products in the photocatalytic CO_2_ reduction processes of CN, Ag/CN-1, and Ag/CN-2. The CH_4_ and CO evolution rates of Ag/CN-1 and Ag/CN-2 are higher than those of CN, indicating that the loading of Ag nanoparticles enhanced the photocatalytic CO_2_ reduction performance. The CH_4_ and CO evolution rates of Ag/CN-2 are up to 10.44 and 88.79 µmol·h^−1^·g^−1^, respectively, which are higher than those of CN and Ag/CN-1. The CH_4_ and CO evolution rates of CN are 4.17 and 35.51 µmol·h^−1^·g^−1^, respectively. The CH_4_ and CO evolution rates of g Ag/CN-1 are 6.26 and 53.27 µmol·h^−1^·g^−1^, respectively. The CH_4_ evolution rates of Ag/CN-2 are 2.5 and 1.7 times higher than those of CN and Ag/CN-1, respectively. The CO evolution rates of Ag/CN-2 are 2.5 and 1.7 times higher than those of CN and Ag/CN-1, respectively. The CH_4_ and CO evolution rates of Ag/CN-2 grow nearly linearly with time (Figure 2b,c) and are much higher than those of CN and Ag/CN-1 during the photothermal catalytic CO_2_ reduction process. Compared with g-CN and Ag/CN-1, Ag/CN-2 exhibits excellent photothermal catalytic CO_2_ reduction performance. The photothermal catalytic CO_2_ reduction performance of Ag/CN-2 is higher than that of many other g-C_3_N_4_-based photocatalytic materials under similar conditions (Table S2, Supporting Information). For instance, the CO evolution rates of Na_3_PO_4_/g-C_3_N_4_ and coral tubular g-C_3_N_4_ are 7.33 and 5.38 µmol·h^−1^·g^−1^, respectively. The CH_4_ and CO yield of Ag/CN-2 during the cycled stable test (Figure 2d) and the XRD patterns hardly change before and after the stability tests involving Ag/CN-2 (Figure S2), indicating that the structure of Ag/CN-2 remains stable during the photothermal catalytic CO_2_ reduction.

From Figure 2e–g, the local temperatures of Ag/CN-1 (53.1 °C) and Ag/CN-2 (70.6 °C) reaction systems are higher than that of CN (46.3 °C). The higher reaction temperatures of Ag/CN-1 and Ag/CN-2 in comparison to that of CN imply that Ag nanoparticles increase visible light absorption and generate hot electrons by LSPR, thereby increasing the local reaction temperature. Thus, the enhanced CH_4_ and CO evolution rates of Ag/CN-2 could be related to the hot electrons induced by the LSPR of Ag nanoparticles. The CH_4_ and CO evolution rates of Ag/CN-2 indicate that the efficient hot electron transfer in the Ag/g-C_3_N_4_ Schottky junction improves photocatalytic CO_2_ reduction activity.

### 3.3. Hot Electron Transfer Route

**Figure 3:**
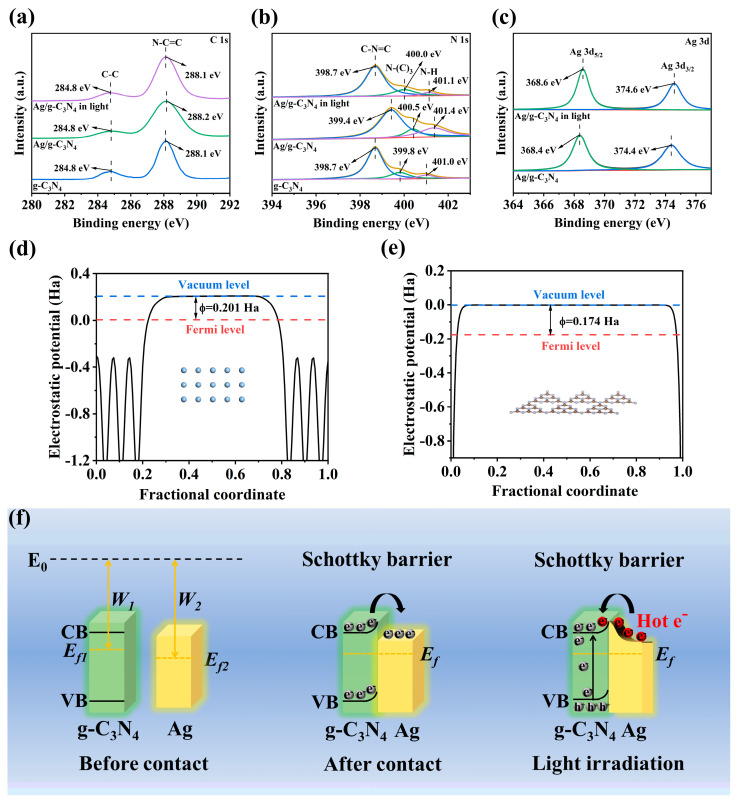
In situ high-resolution XPS spectra of (**a**) C 1s, (**b**) N 1s, and (**c**) Ag 3d of g-C_3_N_4_ and Ag/g-C_3_N_4_; work functions of (**d**) Ag and (**e**) g-C_3_N_4_; (**f**) schematic illustrations of electron transfer mechanism between g-C_3_N_4_ and Ag before contact, after contact, and after contact under light irradiation.

In order to investigate the mechanism of hot electrons induced by localized surface plasmon resonance in the Ag/g-C_3_N_4_ Schottky junction for photothermal catalytic CO_2_ reduction, X-ray photoelectron spectroscopy (XPS) was applied to identify the electronic chemical states and electron transfer of the photocatalyst. The XPS spectra show that C, N, and Ag elements can be detected in the Ag/g-C_3_N_4_, indicating the successful construction of the Ag/g-C_3_N_4_ Schottky junction. As presented in Figure 3a, the peaks for the g-C_3_N_4,_ located at 284.8 and 288.1 eV, can be attributed to C-C and N-C=C of C, respectively. Furthermore, the peaks centered at 398.7, 399.8, and 401.0 eV are assigned to C-N=C, N-(C)_3,_, and C-N-H, respectively (Figure 3b) [29,43]. In general, the change in electron binding energy reflects the change in electron density. Therefore, the change in electron binding energy can verify the direction of electron transfer in the Schottky junction photocatalyst [44,45,46]. As Figure 3a,b show, the binding energies of C-C and N-C=C in Ag/g-C_3_N_4_ shift toward higher energy levels, indicating that g-C_3_N_4_ loses electrons. Meanwhile, the binding energy of C-N=C, N-(C)_3,_, and C-N-H in Ag/g-C_3_N_4_ shifts to a higher energy level, implying that g-C_3_N_4_ loses electrons. The peaks centered at 368.4 and 374.4 eV are assigned to Ag 3d5/2 and Ag 3d3/2 in Ag/g-C_3_N_4_, respectively (Figure 3c) [47,48]. The electrons transfer from g-C_3_N_4_ to Ag in the Ag/g-C_3_N_4_ Schottky junction. In contrast, the binding energies of C 1s and N 1s in Ag/g-C_3_N_4_ shift toward lower energy levels upon light irradiation, while the binding energies of Ag 3d shift to a higher energy level. The in situ high-resolution XPS spectra indicate that the hot electrons transfer from Ag to g-C_3_N_4_. Therefore, the electrons firstly transfer from g-C_3_N_4_ to Ag, and the hot electrons migrate from Ag to g-C_3_N_4_ in the Ag/g-C_3_N_4_ Schottky junction upon irradiation.

Based on the results of in situ irradiated XPS, the electron and hot electron transfer routes between g-C_3_N_4_ and Ag in the Ag/g-C_3_N_4_ Schottky junction are summarized. The work functions (Φ) of Ag and g-C_3_N_4_ were calculated using density functional theory (DFT). Figure 3d,e show that the work function of g-C_3_N_4_ (0.174 Ha) is smaller than that of Ag (0.201 Ha), which indicates that the Fermi level of Ag is lower than that of g-C_3_N_4_. As shown in Figure 3f, before the contact of Ag and g-C_3_N_4_, g-C_3_N_4_ has a smaller W_1_ and higher E_f1_, while Ag has a larger W_2_ and lower E_f2_. The difference in work function will lead to band bending at the interface between Ag and g-C_3_N_4_. After Ag makes contact with g-C_3_N_4_, driven by band bending, the electrons transfer from the g-C_3_N_4_ to the Ag. When the Ag/g-C_3_N_4_ Schottky junction is formed upon irradiation, the electrons are excited from the VB to the CB of g-C_3_N_4_, and hot electrons are generated at the surface of Ag. Then, the hot electrons can overstep the Schottky barrier and transfer from Ag into the CB of g-C_3_N_4_ at the interface because hot electrons possess higher energies than those of normally excited electrons, leading to the efficient separation and transfer of photogenerated electron–hole pairs in g-C_3_N_4_. The efficient separation and transfer of photogenerated electron–hole pairs in the Ag/g-C_3_N_4_ Schottky junction improved the photocatalytic CO_2_ reduction performance.

**Figure 4:**
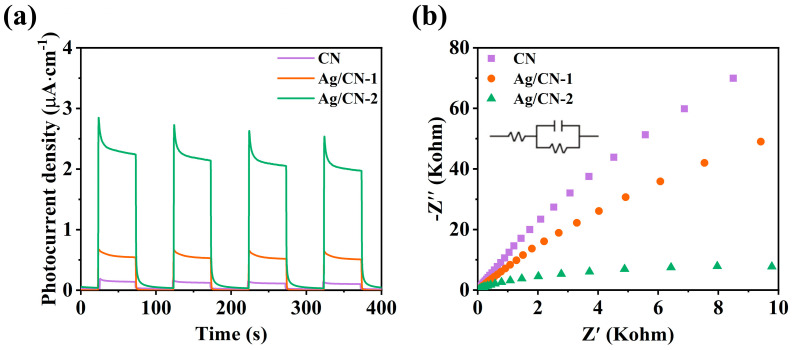
(**a**) Transient photocurrent spectra (TPC); (**b**) electrochemical impedance spectroscopy (EIS) spectra of CN, Ag/CN-1, and Ag/CN-2.

Electrochemical impedance is the electrical resistance that occurs during charge carrier transport in photocatalysts [42]. The photocurrent in semiconductors is caused by light irradiation. The photons excite the electrons in the valence band of the semiconductor to the conduction band and generate an electric current upon irradiation. Electrochemical impedance spectra (EIS) and transient photocurrent (TPC) spectra usually can be employed to investigate the charge carriers separation and transfer behavior in photocatalysts. As shown in Figure 4a, the photocurrent densities of Ag/CN-1 or Ag/CN-2 are higher than that of CN. Ag/CN-1 and Ag/CN-2 show a smaller electrochemical impedance spectroscopy radius than that of CN (Figure 4b). The TPC and EIS results illustrate that the hot electrons of Ag migrate to g-C_3_N_4,_ improving the efficient photogenerated charge carrier separation and transfer in the Ag/g-C_3_N_4_ Schottky junction.

**Figure 5:**
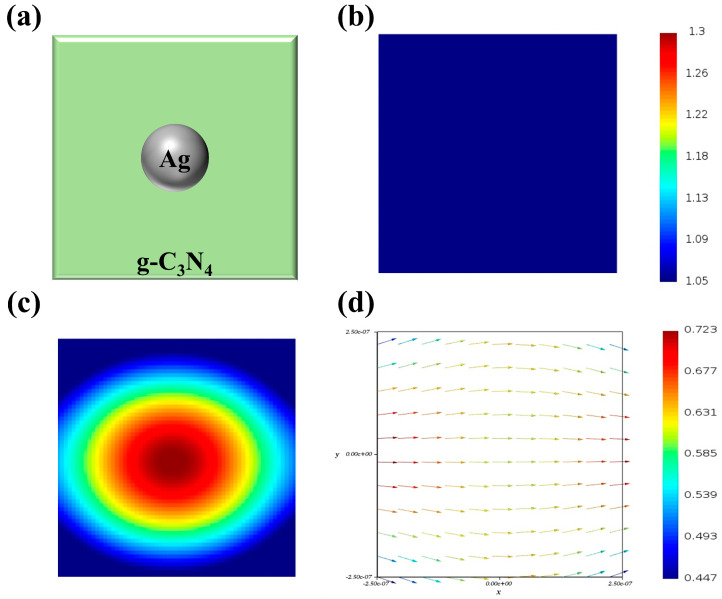
(**a**) FDTD simulation model of Ag/g-C_3_N_4_; electromagnetic fields of (**b**) g-C_3_N_4_ and (**c**) Ag/g-C_3_N_4_; (**d**) electric-field vector of Ag/g-C_3_N_4_ under visible light irradiation.

To better determine the electromagnetic field and electric-field vector of Ag/g-C_3_N_4_, three-dimensional finite-difference time-domain (3D-FDTD) simulations were performed to calculate the spatial electric field distributions as a function of the incident wavelength of light. The FDTD simulation model of Ag/g-C_3_N_4_ is illustrated in Figure 5a. The electromagnetic field of Ag nanoparticle loading at the g-C_3_N_4_ is shown in Figure 5c. The electromagnetic field at the interface between the Ag nanoparticle and the g-C_3_N_4_ is stronger than that of the g-C_3_N_4_ (Figure 5b) under the excitation of visible light, which indicates that more hot electrons have emerged at the interface between the Ag nanoparticle and the g-C_3_N_4_. Figure 5d shows the electric-field vector for Ag nanoparticle loading at g-C_3_N_4_, indicating that electrons have transferred from the TiO_2_ to the Pt nanoparticle; hot electrons could then be generated on the surface of the Ag nanoparticle and transferred from the Ag nanoparticle to the g-C_3_N_4_. The FDTD simulation results are very consistent with the experimental and characterization results.

### 3.4. Photothermal Catalytic CO_2_ Reduction Mechanism

**Figure 6:**
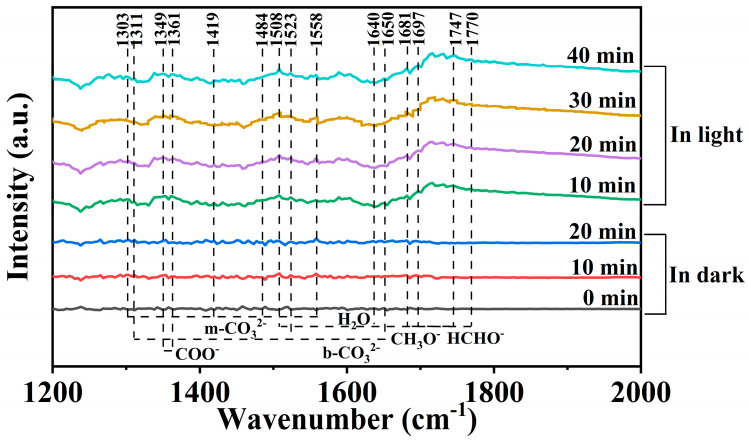
In situ diffuse reflectance infrared Fourier transform spectra (DRIFTS) of Ag/g-C_3_N_4_.

In situ diffuse reflectance infrared Fourier transform spectroscopy (DRIFTS) spectra were applied to investigate the photothermal catalytic CO_2_ reduction reaction pathway and possible reaction carbon intermediates on the surface of the Ag/g-C_3_N_4_ photocatalyst. As shown in Figure 6, DRIFTS spectra of Ag/g-C_3_N_4_ in dark and light were collected in the photothermal catalytic CO_2_ reduction process. Initially, no absorption peaks can be detected in the spectrum of the Ag/g-C_3_N_4_ photocatalyst in the dark (0 min). With the introduction of CO_2_ and H_2_ (10–20 min), monodentate carbonate species (m-CO_3_^2−^, 1303, 1419, 1484, and 1558 cm^−1^) and bidentate carbonate species (b-CO_3_^2−^, 1311,1523, and 1650 cm^−1^) appear in the spectra [49,50,51,52]. The signals of m-CO_3_^2−^ and b-CO_3_^2−^ in the spectra indicate that CO_2_ was successfully adsorbed and activated on the surface of Ag/g-C_3_N_4_. New signal peaks appear in the spectra upon irradiation (10–40 min). The carboxylate species (COO^−^, 1349 and 1361 cm^−1^), methoxy groups (CH_3_O^−^, 1681, 1697 and 17,471 cm^−1^), and formaldehyde species (HCHO^−^, 1508 and 1770 cm^−1^) are detected in the photothermal catalytic CO_2_ reduction of Ag/g-C_3_N_4_ [49,51,53]. The COO^−^, CH_3_O^−^, and HCHO^−^ species are crucial reaction intermediates in the formation of CH_4_ and CO. Therefore, the proposed reaction pathways of photothermal catalytic CO_2_ reduction to CH_4_ and CO over the Ag/g-C_3_N_4_ photocatalyst can be briefly expressed as follows: CO_2_ → COO^−^ → CO and CO_2_ → COO^−^ → HCHO^−^ → CH_3_O^−^ → CH_4_.

**Figure 7:**
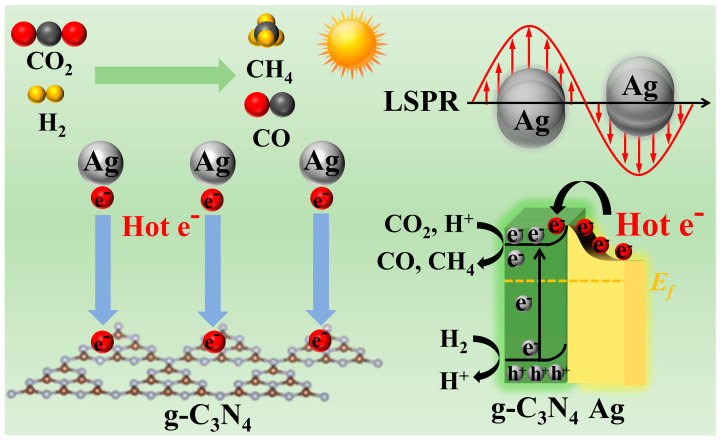
Schematic diagram of photothermal catalytic CO_2_ reduction over Ag/g-C_3_N_4_.

Thus, the probable mechanism of the Ag/g-C_3_N_4_ Schottky junction for photothermal catalytic CO_2_ reduction can be proposed (Figure 7). Under light irradiation, photogenerated electrons are excited from the valence band to the conduction band of g-C_3_N_4_, while hot electrons are generated at the surface of Ag due to LSPR. Then, hot electrons at the surface of Ag can overstep the Schottky barrier and transfer from Ag to the CB of g-C_3_N_4_, promoting the separation and transfer of photogenerated electron–hole pairs in the Ag/g-C_3_N_4_ Schottky junction. Meanwhile, H_2_ is oxidized into H^+^ on the VB, and CO_2_ is reduced into CH_4_ and CO on the CB. Additionally, the transfer of hot electrons from Ag to g-C_3_N_4_ improves the local reaction temperature, thereby enhancing the photocatalytic CO_2_ reduction performance. The efficient hot electron transfer in the Ag/g-C_3_N_4_ Schottky junction enhances the photocatalytic CO_2_ reduction performance.

## 4. Conclusions

In summary, the photothermal catalytic CO_2_ reduction performance could be enhanced by the Ag/g-C_3_N_4_ Schottky junction photocatalyst featuring LSPR. Compared with g-C_3_N_4_, due to the efficient hot electron transfer in the Schottky junction, the photocatalytic CO_2_ reduction performance of Ag/g-C_3_N_4_ was significantly enhanced. The electron and hot electron transfer mechanisms in the Ag/g-C_3_N_4_ Schottky junction were proven using in situ irradiated XPS. The hot electrons migrate from Ag to g-C_3_N_4_, improving the local reaction temperature and promoting the separation and transfer of photogenerated electron–hole pairs in the Ag/g-C_3_N_4_ Schottky junction. The 3D-FDTD assessment verified the strong electromagnetic field at the interface between Ag and g-C_3_N_4_ and the generation of hot electrons on Ag nanoparticles. The photothermal catalytic reduction of CO_2_ to CH_4_ and CO pathways on the Ag/g-C_3_N_4_ was verified using in situ DRIFTS. The photothermal catalytic reduction of CO_2_ to CO and CH_4_ pathways over Ag/g-C_3_N_4_ is briefly expressed as CO_2_ → COO^−^ → CO and CO_2_ → COO^−^ → HCHO^−^ → CH_3_O^−^ → CH_4_. Therefore, this work reveals the hot electron transfer route and photothermal catalytic CO_2_ reduction reaction pathway in the Ag/g-C_3_N_4_ Schottky junction and provides a feasible approach to designing a plasmonic metal/polymer semiconductor Schottky junction for photothermal catalytic CO_2_ reduction.

## Data Availability

The original contributions presented in the study are included in the article/Supplementary Material, further inquiries can be directed to the corresponding authors.

## Supplementary Materials

The following supporting information can be downloaded at: https://www.mdpi.com/article/10.3390/polym16162317/s1; Figure S1: Nitrogen adsorption-desorption isotherm of CN, Ag/CN-1, and Ag/CN-2; Figure S2: XRD patterns before and after the stability tests of Ag/CN-2; Figure S3: DFT calculation model of g-C_3_N_4_; Figure S4: DFT calculation model of Ag; Table S1: Brunauer–Emmett–Teller surface areas (S_BET_) of samples; Table S2: Performance comparison of g-C_3_N_4_-based photocatalytic materials for CO_2_ reduction. References [54,55,56,57] are cited in the supplementary materials.

## References

[1] Barral A., Gomez B., Fourel F., Daviero-Gomez V., Lécuyer C. (2017). CO_2_ and temperature decoupling at the million-year scale during the Cretaceous Greenhouse. Sci. Rep..

[2] Nordt L., Breecker D., White J. (2022). Jurassic greenhouse ice-sheet fluctuations sensitive to atmospheric CO_2_ dynamics. Nat. Geosci..

[3] Ortega T., Jiménez-López D., Sierra A., Ponce R., Forja J. (2023). Greenhouse gas assemblages (CO_2_, CH_4_ and N_2_O) in the continental shelf of the Gulf of Cadiz (SW Iberian Peninsula). Sci. Total. Environ..

[4] Chen B., Tan E., Zou W., Han L.-L., Tian L., Kao S.-J. (2024). The external/internal sources and sinks of greenhouse gases (CO_2_, CH_4_, N_2_O) in the Pearl River Estuary and adjacent coastal waters in summer. Water Res..

[5] Fang S., Rahaman M., Bharti J., Reisner E., Robert M., Ozin G.A., Hu Y.H. (2023). Photocatalytic CO_2_ reduction. Nat. Rev. Methods Primers.

[6] Albero J., Peng Y., García H. (2020). Photocatalytic CO_2_ Reduction to C2+ Products. ACS Catal..

[7] Qu T., Wei S., Xiong Z., Zhang J., Zhao Y. (2023). Progress and prospect of CO_2_ photocatalytic reduction to methanol. Fuel Process. Technol..

[8] Low J., Zhang C., Karadas F., Xiong Y. (2023). Photocatalytic CO_2_ conversion: Beyond the earth. Chin. J. Catal..

[9] Gao M., Zhang T., Ho G.W. (2022). Advances of photothermal chemistry in photocatalysis, thermocatalysis, and synergetic photothermocatalysis for solar-to-fuel generation. Nano Res..

[10] Xiao J.-D., Jiang H.-L. (2019). Metal–Organic Frameworks for Photocatalysis and Photothermal Catalysis. Accounts Chem. Res..

[11] Fresno F., Iglesias-Juez A., Coronado J.M. (2023). Photothermal Catalytic CO_2_ Conversion: Beyond Catalysis and Photocatalysis. Top. Curr. Chem..

[12] Xi Y., Du C., Li P., Zhou X., Zhou C., Yang S. (2022). Combination of Photothermal Conversion and Photocatalysis toward Water Purification. Ind. Eng. Chem. Res..

[13] Chen Y., Fang J., Dai B., Kou J., Lu C., Xu Z. (2020). Photothermal effect enhanced photocatalysis realized by photonic crystal and microreactor. Appl. Surf. Sci..

[14] Lu Y., Zhang H., Fan D., Chen Z., Yang X. (2022). Coupling solar-driven photothermal effect into photocatalysis for sustainable water treatment. J. Hazard. Mater..

[15] Sun D., Joo J.-U., Kim D.-P. (2023). Photothermally accelerated photocatalysis over hollow carbon@ZnIn2S4 for enhanced amine oxidation. React. Chem. Eng..

[16] Jiang P., Zhou L., Han Y., Fu W., Su S., Zeng M. (2024). Utilizing waste corn straw to photodegrade methyl orange and methylene blue: Photothermal effect of biochar enhances photodegradation efficiency. J. Environ. Chem. Eng..

[17] Becker H., Ziegenbalg D., Güttel R. (2023). Discriminating photochemical and photothermal effects in heterogeneous photocatalysis. Catal. Sci. Technol..

[18] Chen X., Wu H., Shi X., Wu L. (2023). Polyoxometalate-based frameworks for photocatalysis and photothermal catalysis. Nanoscale.

[19] Xi Y., Cai M., Wu Z., Zhu Z., Shen J., Zhang C., Tang R., An X., Li C., He L. (2023). Identification of photochemical effects in Ni-based photothermal catalysts. Chin. J. Struct. Chem..

[20] Sun Z., Huang X., Zhang G. (2022). TiO_2_-based catalysts for photothermal catalysis: Mechanisms, materials and applications. J. Clean. Prod..

[21] Zhang F., Li Y.-H., Qi M.-Y., Yamada Y.M.A., Anpo M., Tang Z.-R., Xu Y.-J. (2021). Photothermal catalytic CO_2_ reduction over nanomaterials. Chem. Catal..

[22] Guene Lougou B., Geng B.-X., Pan R.-M., Wang W., Yan T.-T., Li F.-H., Zhang H., Djandja O.S., Shuai Y., Tabatabaei M. (2024). Solar-driven photothermal catalytic CO_2_ conversion: A review. Rare Met..

[23] Geng Z., Yu Y., Liu J. (2023). Broadband Plasmonic Photocatalysis Enhanced by Photothermal Light Absorbers. J. Phys. Chem. C.

[24] Ge H., Kuwahara Y., Yamashita H. (2022). Development of defective molybdenum oxides for photocatalysis, thermal catalysis, and photothermal catalysis. Chem. Commun..

[25] Wan Y., Liu Q., Xu Z., Li J., Wang H., Xu M., Yan C., Song X., Liu X., Wang H. (2024). Interface engineering enhanced g-C_3_N_4_/rGO/Pd composites synergetic localized surface plasmon resonance effect for boosting photocatalytic CO_2_ reduction. Carbon Lett..

[26] Liu T., Tan G., Feng S., Zhang B., Liu Y., Wang Z., Bi Y., Yang Q., Xia A., Liu W. (2023). Dual Localized Surface Plasmon Resonance effect enhances Nb_2_AlC/Nb_2_C MXene thermally coupled photocatalytic reduction of CO_2_ hydrogenation activity. J. Colloid. Interf. Sci..

[27] Ge H., Kuwahara Y., Kusu K., Bian Z., Yamashita H. (2022). Ru/HxMoO_3_-y with plasmonic effect for boosting photothermal catalytic CO_2_ methanation. Appl. Catal. B Environ..

[28] Li J., Xu Q., Han Y., Guo Z., Zhao L., Cheng K., Zhang Q., Wang Y. (2023). Efficient photothermal CO_2_ methanation over NiFe alloy nanoparticles with enhanced localized surface plasmon resonance effect. Sci. China Chem..

[29] Wang L., Dong Y., Zhang J., Tao F., Xu J. (2022). Construction of NiO/g-C_3_N_4_ p-n heterojunctions for enhanced photocatalytic CO_2_ reduction. J. Solid State Chem..

[30] Ye L., Wu D., Chu K.H., Wang B., Xie H., Yip H.Y., Wong P.K. (2016). Phosphorylation of g-C_3_N_4_ for enhanced photocatalytic CO_2_ reduction. Chem. Eng. J..

[31] Sun Z., Wang H., Wu Z., Wang L. (2018). g-C_3_N_4_ based composite photocatalysts for photocatalytic CO_2_ reduction. Catal. Today.

[32] Ghosh U., Majumdar A., Pal A. (2021). Photocatalytic CO_2_ reduction over g-C_3_N_4_ based heterostructures: Recent progress and prospects. J. Environ. Chem. Eng..

[33] Li J., Lou Z., Li B. (2022). Nanostructured materials with localized surface plasmon resonance for photocatalysis. Chin. Chem. Lett..

[34] Wang J., Jin M., Gong Y., Li H., Wu S., Zhang Z., Zhou G., Shui L., Eijkel J.C.T., van den Berg A. (2017). Continuous fabrication of microcapsules with controllable metal covered nanoparticle arrays using droplet microfluidics for localized surface plasmon resonance. Lab A Chip.

[35] Lee H., Song K., Lee M., Park J.Y. (2020). In Situ Visualization of Localized Surface Plasmon Resonance-Driven Hot Hole Flux. Adv. Sci..

[36] Zheng L., Yang Y., Bowen C.R., Jiang L., Shu Z., He Y., Yang H., Xie Z., Lu T., Hu F. (2023). A high-performance UV photodetector with superior responsivity enabled by a synergistic photo/thermal enhancement of localized surface plasmon resonance. J. Mater. Chem. C.

[37] Wang D., Huang L., Guo Z., Han X., Liu C., Wang W., Yuan W. (2018). Enhanced photocatalytic hydrogen production over Au/SiC for water reduction by localized surface plasmon resonance effect. Appl. Surf. Sci..

[38] Zhao S., Yin Y., Peng J., Wu Y., Andersson G.G., Beck F.J. (2021). The Importance of Schottky Barrier Height in Plasmonically Enhanced Hot-Electron Devices. Adv. Optical. Mater..

[39] Jeon B., Lee C., Park J.Y. (2021). Electronic Control of Hot Electron Transport Using Modified Schottky Barriers in Metal–Semiconductor Nanodiodes. ACS Appl. Mater. Interf..

[40] Feng Y., Wang C., Cui P., Li C., Zhang B., Gan L., Zhang S., Zhang X., Zhou X., Sun Z. (2022). Ultrahigh Photocatalytic CO_2_ Reduction Efficiency and Selectivity Manipulation by Single-Tungsten-Atom Oxide at the Atomic Step of TiO_2_. Adv. Mater..

[41] Wang Y., Yang W., Chen X., Wang J., Zhu Y. (2018). Photocatalytic activity enhancement of core-shell structure g-C_3_N_4_@TiO_2_ via controlled ultrathin g-C_3_N_4_ layer. Appl. Catal. B Environ..

[42] Rahmati M., Mohammadi Zahrani E., Atapour M., Noorbakhsh Nezhad A.H., Hakimizad A., Alfantazi A.M. (2024). In situ synthesis and electrochemical corrosion behavior of plasma electrolytic oxidation coating containing an osteoporosis drug on AZ31 magnesium alloy. Mater. Chem. Phys..

[43] Jiang P., Yu Y., Wang K., Liu W. (2024). Efficient Electron Transfer in g-C_3_N_4_/TiO_2_ Heterojunction for Enhanced Photocatalytic CO_2_ Reduction. Catalysts.

[44] Low J., Dai B., Tong T., Jiang C., Yu J. (2019). In Situ Irradiated X-Ray Photoelectron Spectroscopy Investigation on a Direct Z-Scheme TiO_2_/CdS Composite Film Photocatalyst. Adv. Mater..

[45] Wang L., Cheng B., Zhang L., Yu J. (2021). In situ Irradiated XPS Investigation on S-Scheme TiO_2_@ZnIn_2_S_4_ Photocatalyst for Efficient Photocatalytic CO_2_ Reduction. Small.

[46] Zhang P., Li Y., Zhang Y., Hou R., Zhang X., Xue C., Wang S., Zhu B., Li N., Shao G. (2020). Photogenerated Electron Transfer Process in Heterojunctions: In Situ Irradiation XPS. Small Methods.

[47] Li Y., Yin W., Li M., Zhang J., Chen L. (2022). Multi-component Ag/AgCl/Bi_2_O_3_/BiFeO_3_ for the sunlight-induced photocatalytic degradation. J. Environ. Chem. Eng..

[48] Phu N.D., Hoang L.H., Van Hai P., Huy T.Q., Chen X.-B., Chou W.C. (2020). Photocatalytic activity enhancement of Bi_2_WO_6_ nanoparticles by Ag doping and Ag nanoparticles modification. J. Alloys Compd..

[49] Bie C., Zhu B., Xu F., Zhang L., Yu J. (2019). In Situ Grown Monolayer N-Doped Graphene on CdS Hollow Spheres with Seamless Contact for Photocatalytic CO_2_ Reduction. Adv. Mater..

[50] Deng Y., Wan C., Li C., Wang Y., Mu X., Liu W., Huang Y., Wong P.K., Ye L. (2022). Synergy Effect between Facet and Zero-Valent Copper for Selectivity Photocatalytic Methane Formation from CO_2_. ACS Catal..

[51] He F., Zhu B., Cheng B., Yu J., Ho W., Macyk W. (2020). 2D/2D/0D TiO_2_/C_3_N_4_/Ti_3_C_2_ MXene composite S-scheme photocatalyst with enhanced CO_2_ reduction activity. Appl. Catal. B Environ..

[52] Yin G., Huang X., Chen T., Zhao W., Bi Q., Xu J., Han Y., Huang F. (2018). Hydrogenated Blue Titania for Efficient Solar to Chemical Conversions: Preparation, Characterization, and Reaction Mechanism of CO_2_ Reduction. ACS Catal..

[53] Wang L., Tan H., Zhang L., Cheng B., Yu J. (2021). In-situ growth of few-layer graphene on ZnO with intimate interfacial contact for enhanced photocatalytic CO_2_ reduction activity. Chem. Eng. J..

[54] Wu Q., Jiang H., Ren H., Wu Y., Zhou Y., Chen J., Xu X., Wu X. (2024). Surface C triple bond N bonds mediate photocatalytic CO_2_ reduction into efficient CH_4_ production in TiO_2_-decorated g-C_3_N_4_ nanosheets. J. Colloid. Interf. Sci..

[55] Li Z., Ao J., Wang Z., Huang Z., Xu Z., Wu X., Cheng Z., Lv K. (2024). Boosting the photocatalytic CO_2_ reduction activity of g-C_3_N_4_ by acid modification. Sep. Purif. Technol..

[56] Xu X., Huang Y., Dai K., Wang Z., Zhang J. (2023). Non-noble-metal CuSe promotes charge separation and photocatalytic CO_2_ reduction on porous g-C_3_N_4_ nanosheets. Sep. Purif. Technol..

[57] Jia Y., Tong X., Zhang J., Zhang R., Yang Y., Zhang L., Ji X. (2023). A facile synthesis of coral tubular g-C_3_N_4_ for photocatalytic degradation RhB and CO_2_ reduction. J. Alloy. Compd..

