# Supplementary material for: Hot Electrons Induced by Localized Surface Plasmon Resonance in Ag/g-C3N4 Schottky Junction for Photothermal Catalytic CO2 Reduction
Source: Polymers (Basel). 2024 Aug 16;16(16):2317. doi: 10.3390/polym16162317 (PMC11359675; doi:10.3390/polym16162317)
Supplement: Supplementary file 1 [file polymers-16-02317-s001.zip › polymers-2961438-supplementary.pdf]

Supporting Information

Hot electrons induced by Localized Surface Plasmon Resonance in $Ag/g\text{-}C_3N_4$ Schottky Junction for Photothermal catalytic CO_2 Reduction

Peng Jiang[1], Kun Wang[1], Wenrui Liu[1], Yuhang Song[1], Runtian Zheng[2], Lihua Chen[1,*], Baolian Su[1,2,*]

1 State Key Laboratory of Advanced Technology for Materials Synthesis and Processing, Wuhan University of Technology, Wuhan 430070, China

2 Laboratory of Inorganic Materials Chemistry, University of Namur, B-5000 Namur, Belgium

*Correspondence: chenlihua@whut.edu.cn (Li-Hua Chen); bao-lian.su@unamur.be (Bao-Lian Su)

Characterization

The microstructures were observed by FSEM (Hitach S-4800, Japan) and TEM (Talos F200S, USA). The XRD patterns were obtained by an X-ray diffraction machine (Bruker D8 Advance, Germany) using Cu Kα (λ = 0.15418 nm) radiation. The X-ray photoelectron spectroscopy (XPS, Omicron Sphera II, Germany) data was performed on a mono-chromated Al Kα Xray source (hv=1486.6 eV) at 15 kV/150 W to detect the chemical states of elements in the samples. The XRD analysis's time per step is 0.5 degrees and the step size is 10 to 80 degrees. The UV–vis diffuse reflectance spectra (UV–vis DRS) were acquired by UV–VIS-NIR spectrometer (Lambda 750S, PerkinElmer, USA) over a range of 200–800 nm. Transient photocurrent spectra (TPC) and electrochemical impedance spectroscopy (EIS) were measured on an electrochemical workstation (CS2350H, CorrTest, China) in 0.5 M Na_2SO_4 solution at room temperature using a 300W Xenon lamp (PLS-SXE300, PerfectLight, China). The potential is 0.5 eV and the frequency range is 0 to 100000 Hz. The Pt plate and Ag/AgCl were employed as the counter electrode and the reference electrode, respectively. The surface area of the working electrode is 1 cm^2. The working electrode was prepared by coating a catalyst on a conductive glass. In-situ time-resolved DRIFT spectra were obtained by a Fourier transform infrared spectrometer (VERTEX V80, Bruker, Germany). Infrared thermograms were taken by Infrared Thermal Camera (UTi260B, China). The work functions of Ag and g-C_3N_4 were calculated by DFT in Materials Studio. The finite-difference time-domain (FDTD) simulations were performed by Lumerical Solutions. The details of DFT calculations and FDTD simulations are listed in the supporting information.

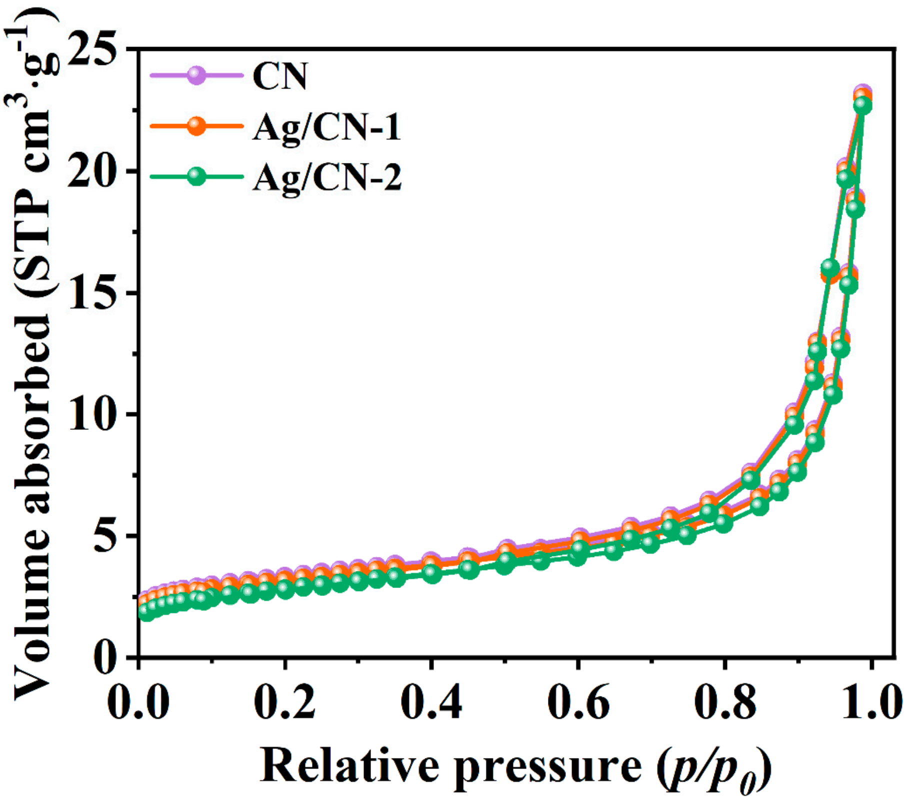

Figure S1. Nitrogen adsorption–desorption isotherm of CN, Ag/CN-1 and Ag/CN-2.

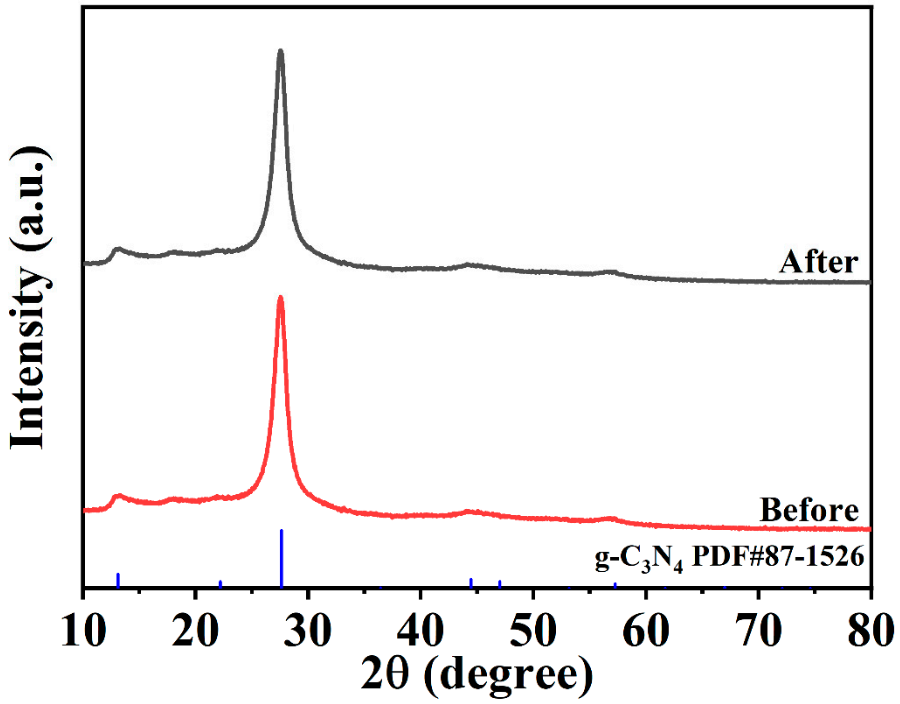

Figure S2. XRD patterns before and after the stability tests of Ag/CN-2.

Table S1. Brunauer–Emmett–Teller surface areas (S_{BET}) of samples.

Catalyst	S_{BET} ($m^2·g^{-1}$)
CN	13.5
Ag/CN-1	12.2
Ag/CN-2	11.4

Table S2. Performance comparison of g-C_3N_4-based photocatalytic materials for CO_2 reduction.

Photocatalysis	Product	Yield ($\mu mol·g^{-1}·h^{-1}$)	Reference
Ag/CN-2	CH_4	10.44	This work
	CO	88.79	
MCN/TiO_2	CH_4	26.59	1
	CO	3.90	
Na_3PO_4/g-C_3N_4	CO	7.33	2
CuSe/g-C_3N_4	CO	25.03	3
Coral tubular g-C_3N_4	CO	5.38	4

DFT Calculation

The work functions of g-C_3N_4 and Ag were calculated by density functional theory (DFT) in Materials Studio. DFT calculation was performed by using the CASTEP module. The exchange-correlation interaction was described by generalized gradient approximation (GGA) with the Perdew–Burke–Ernzerhof (PBE) functional. The energy cutoff was set to 570 eV. The Monkhorst-Pack k-point mesh was set as 3 × 3 × 1. A vacuum space with a thickness of 20 Å was used to eliminate interactions between periodic structures.

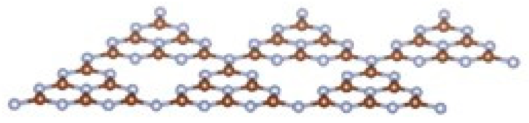

Figure S3. DFT calculation model of g-C_3N_4.

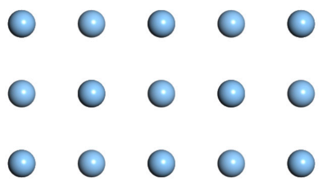

Figure S4. DFT calculation model of Ag.

Three-dimensional Finite Difference Time Domain (3D-FDTD)

The 3D-FDTD method was employed to study the electric field intensity and Electric-field vector of Ag/g-C_3N_4 (FDTD Solutions 8.19.1584 Lumerical Solutions, Inc.). The electromagnetic analysis is performed by solving Maxwell's equations:

$$\nabla \times \mathbf{H} = \mathbf{J} + \frac{\partial \mathbf{D}}{\partial t} \tag{1}$$

$$\nabla \times \mathbf{E} = -\frac{\partial \mathbf{B}}{\partial t} \tag{2}$$

$$\nabla \cdot \mathbf{D} = \rho \tag{3}$$

$$\nabla \cdot \mathbf{B} = 0 \tag{4}$$

where **E** is the electric field intensity; **D** is the electric displacement; **H** is the magnetic field intensity; **B** is the magnetic flux density; **J** is the current density, and **ρ** is the electric charge density. To obtain a closed system, the constitutive relations of the material properties must be included. They are given as follows:

$$\mathbf{D} = \varepsilon \mathbf{E} \tag{5}$$

$$\mathbf{B} = \mu \mathbf{H} \tag{6}$$

$$\mathbf{J} = \sigma \mathbf{E} \tag{7}$$

where ε and μ are the permittivity and permeability of the material, respectively; σ is the conductivity of the material. By solving the Maxwell's equations, electric field intensity and Electric-field vector are obtained.

References

1.Wu, Q.; Jiang, H.; Ren, H.; Wu, Y.; Zhou, Y.; Chen, J.; Xu, X.; Wu, X. Surface C triple bond N bonds mediate photocatalytic CO_2 reduction into efficient CH_4 production in TiO_2-decorated g-C_3N_4 nanosheets. J. Colloid. Interf. Sci. **2024**, 663, 825-833.

2. Li, Z.; Ao, J.; Wang, Z.; Huang, Z.; Xu, Z.; Wu, X.; Cheng, Z.; Lv, K.; Boosting the photocatalytic CO_2 reduction activity of g-C_3N_4 by acid modification. Sep. Purif. Technol. **2024**, 338, 126577.

3. Xu, X.; Huang, Y.; Dai, K.; Wang, Z.; Zhang, J. Non-noble-metal CuSe promotes charge separation and photocatalytic CO_2 reduction on porous g-C_3N_4 nanosheets. **2023**, 317, 123887.

4. Jia, Y.; Tong, X.; Zhang, J.; Zhang, R.; Yang, Y.; Zhang, L.; Ji, X. A facile synthesis of coral tubular g-C_3N_4 for photocatalytic degradation RhB and CO_2 reduction. J. Alloy. Compd. **2023**, 965, 171432.
